# Supplementary material for: Novel Hybrid Inulin–Soy Protein Nanoparticles Simultaneously Loaded with (-)-Epicatechin and Quercetin and Their In Vitro Evaluation
Source: Nanomaterials (Basel). 2023 May 11;13(10):1615. doi: 10.3390/nano13101615 (PMC10223423; doi:10.3390/nano13101615)
Supplement: Supplementary file 1 [file nanomaterials-13-01615-s001.zip › nanomaterials-2363712-supplementary.pdf]

Supplementary Material

Supplementary Table S1. Release exponents in each equation and description of meaning.

Mathematical model		Reference
**Peppas & Korsmeyer**		[21]
Release exponent (n)	Geometry	Drug release mechanism model
0.50<br>0.45<br>0.43	Planar (thin films)<br>Cylinders<br>Spheres	Fickian diffusion
0.50 < n < 1.0<br>0.45 < n < 0.89<br>0.43 < n < 0.85	Planar (thin films)<br>Cylinders<br>Spheres	Anomalous (non-Fickian transport)
1.0<br>0.89<br>0.85	Planar (thin films)<br>Cylinders<br>Spheres	Case Type I transport
n > 1<br>n > 0.89<br>n > 0.85	Planar (thin films)<br>Cylinders<br>Spheres	Super Case Type II transport
**Weibull**		[25]
b = 1<br>b > 1<br>b < 0.35<br>b ~ 0.35 – 0.39<br>0.39 < b < 0.69<br>b ~ 0.69 – 0.75<br>0.75 < b < 1	First-order release obeying Fick's first law of diffusion<br>Indicative of complex release mechanism<br>Highly disordered spaces much different than the percolation cluster<br>Diffusion in the fractal substrate<br>Diffusion in the fractal or disordered substrate<br>Diffusion in normal Euclidian space<br>Diffusion in normal Euclidian substrate with a contribution of another release mechanism	
**Lidner Lippold**		[25]
b ~ negative values<br>b ~ positive values		Without an initial burst effect<br>With an initial burst effect

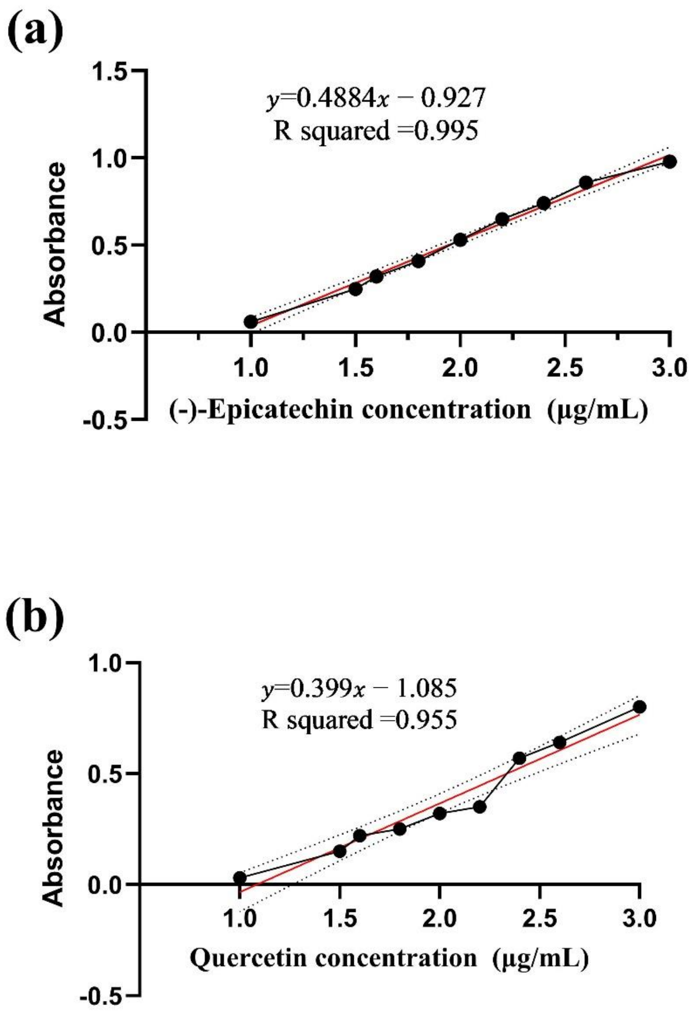

Supplementary Figure S1. Calibration curves for quantification of Released (-)-Epicatechin and Quercetin. The validated HPLC method was used to quantify the released (-)-Epicatechin and Quercetin. Both calibration curves were prepared at pH 7.4 and 37°C. (a) (-)-Epicatechin Calibration Curve: R2=0.995, (b) Quercetin Calibration Curve: R2=0.955.

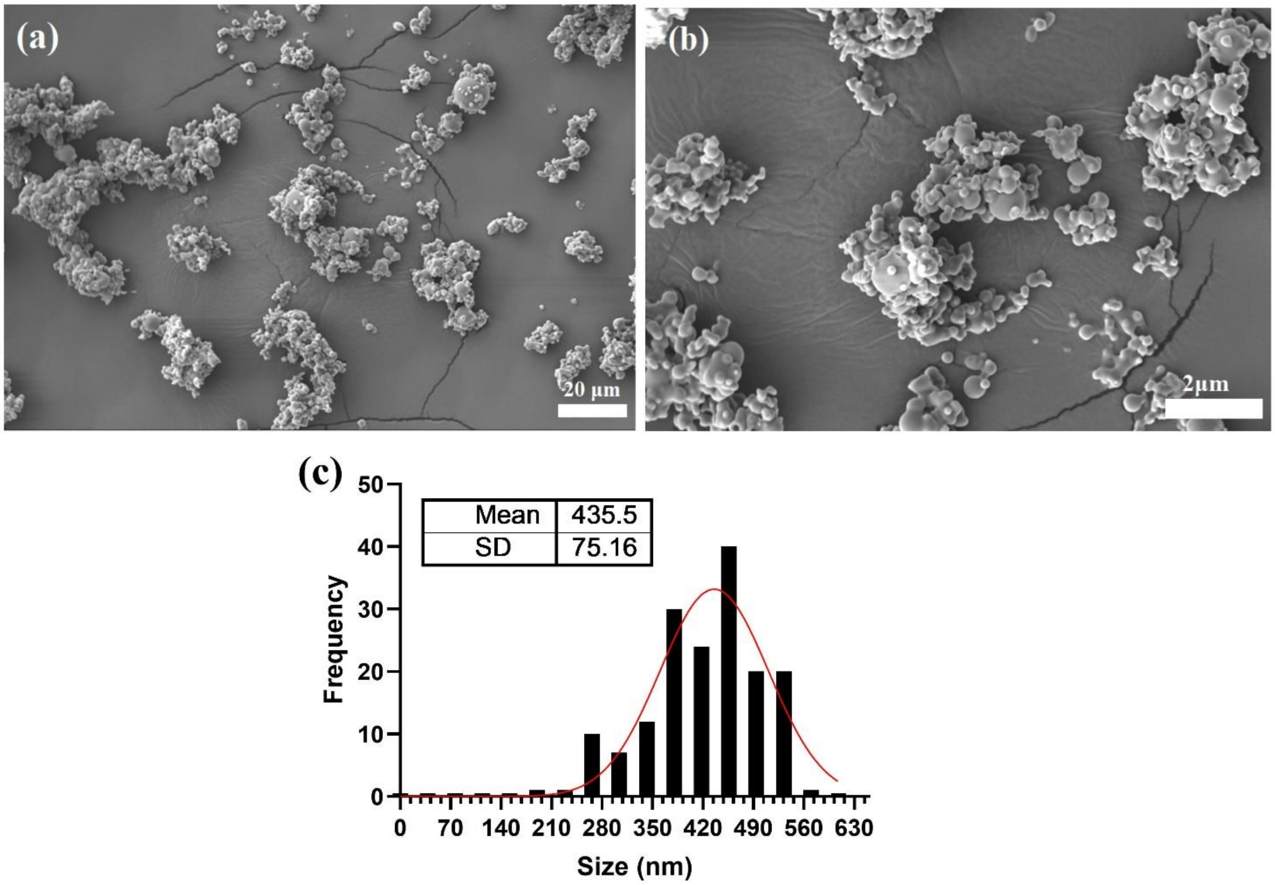

Supplementary Figure S2. Size Characterization by Scanning Electron Microscopy (SEM) of NBPs. (a) and (b) present images of Inulin nanoparticles loaded with flavonoids ((-)- Epicatechin and Quercetin) NBPs. The particle size distribution histograms of NPs (c) were obtained with 165 particles from different areas of the sample, and a non-linear regression of Gaussian type was performed to obtain the average size of the nanoparticles.
